# Effect of smartphone app-based health care intervention for health management of high-risk mothers: a study protocol for a randomized controlled trial

**DOI:** 10.1186/s13063-022-06425-3

**Published:** 2022-06-13

**Authors:** Bora Kim, Jong Youn Moon, Jae Yong Shin, Hae Rin Jeon, So Yeon Oh, Suk Young Kim

**Affiliations:** 1 Department of Preventive Medicine, Gachon University College of Medicine, Incheon, Republic of Korea; 2 Center for Public Health, Gachon University Gil Medical Center, Incheon, 21565 Republic of Korea; 3 Department of Literature and Art Therapy, Konkuk University, Seoul, Republic of Korea; 4 Artificial Intelligence and Big-Data Convergence Center, Gachon University Gil Medical Center, Incheon, 21565 Republic of Korea; 5 Department of Preventive Medicine & Institute of Health Services Research, Yonsei University College of Medicine, Seoul, Republic of Korea; 6 Department of Obstetrics and Gynecology, Gachon University of Gil Medical Center, Incheon, 21565 Republic of Korea; 7 Department of Social & Behavioral Sciences, Harvard TH Chan School of Public Health, Boston, MA USA

**Keywords:** Smartphones apps, High-risk mothers, Healthcare services, Randomized controlled trials, Smart medical technology

## Abstract

**Background:**

The 4th Industrial Revolution with the advent of the smart era, in which artificial intelligence, such as big data analysis and machine learning, is expected, and the provision of healthcare services using smartphones has become a reality. In particular, high-risk mothers who experience gestational diabetes, gestational hypertension, and prenatal and postpartum depression are highly likely to have adverse effects on the mother and newborn due to the disease. Therefore, continuous observation and intervention in health management are needed to prevent diseases and promote healthy behavior for a healthy life.

**Methods:**

This randomized controlled trial will provide mothers 18 years of age or older with health care information collected based on evidence-based literature data using a smartphone app for 6 weeks. About 500 mothers will be selected in consideration of the dropout rate due to the characteristics of mothers. The study group and control group will be computer-generated in a 1:1 ratio through random assignment. The research group will receive health management items through the app, and health management information suitable for the pregnancy cycle is pushed to an alarm. The control group will receive the health management information of the paper. We also followed the procedure for developing mobile apps using the IDEAS framework.

**Discussion:**

These results show the effectiveness of smart medical healthcare services and promote changes in health behaviors throughout pregnancy in high-risk mothers.

**Trial registration:**

Clinical trial registration information for this study has been registered with WHO ICTRP and CRIS (Korea Clinical Research Information Service, CRIS). Clinical trial registration information is as follows:

Study of development of integrated smart health management service for the whole life cycle of high-risk mothers and newborns based on community, KCT0007193. Registered on April 14, 2022, prospectively registered. This protocol version is Version 1.0. April 14, 2022.

## Background

High-risk mothers refer to those who are more likely to have adverse effects on their newborns than normal mothers [1]. They can have a fatal impact on both mothers and newborns because of gestational diabetes, gestational hypertension, and depression during pregnancy. In recent years, medical interventions during pregnancy have shown healthy outcomes for childbirth, mothers, and newborns. Several protocols for high-risk mothers and newborns have been developed abroad through a comparison of normal and high-risk mothers [2]. In the USA, a lifestyle management program is in operation for high-risk mothers with gestational diabetes, and provided guidance and online presentation of lifestyle coaches, modules on diet and exercise management to help women give birth, and information on health and neonatal [3]. In addition, a protocol for collecting maternal healthcare data that can predict and identify patterns of high-risk mothers is also provided; thus, a mother can predict whether she is considered a high-risk mother to prevent any disease progression [4, 5]. In the Netherlands, the Smarter Pregnancy app is provided to prevent pregnancy complications and to manage the health of newborns and mothers [6]. It monitors pregnant women’s questionnaires, blood tests, and physical activity every 18 and 24 weeks, and helps pregnant women manage their hospital appointments and medication adherence. In addition, in Australia, programs and coaching for intervention management of high-risk mothers’ lifestyles, dietary habits, and drinking are provided under the name of “Get Health in Pregnancy,” and it has been confirmed that they manage their mothers through a continuous contact network [7].

However, cognitive-behavioral intervention [8], a psychological theory of behavior, reports that it is important to be able to control and regulate one’s behavior to change it [9, 10]. Based on this, to promote changes to healthy behaviors through psychology and preventive aspects, we propose a behavior change technology [11] that aims for healthy behaviors and provides healthy lifestyle interventions to produce positive results. Therefore, for mothers who need a healthy lifestyle and healthy behavior, services that can provide these are necessary. Studies about using the app, which mediates diet and physical activity of mothers who are overweight and with obesity, were reported to have changes in health behavior [12]. Despite the protocols and healthcare services currently being mediated in many countries to promote healthy behavior for mothers, the fatality rate is high; thus, providing healthcare services for the entire life cycle of high-risk mothers who need more intensive care is important. We believe that the number of protocols used is insignificant [13]. Therefore, providing healthcare services to high-risk mothers throughout the entire pregnancy cycle is vital for a healthier behavior, reducing the maternal mortality rate, the incidence of complications in high-risk mothers and newborns, and improving the survival rate. It is expected to be the most powerful preventive measure against low fertility. Although the health management information protocol is expected to be quite effective for high-risk pregnant mothers, we currently lack the means to solve this problem. Therefore, it is impossible to link the treatments for high-risk mothers and newborns. In addition, as non-face-to-face personal health management culture using ICT is spreading due to COVID-19 with an increasing interest in personal health information, preventive measures, and collection of medical data of high-risk mothers, newborns, and women of childbearing [14] are necessary to improve the quality of life and alleviate the burden of medical expenses through a regular health management system using digital technology.

On the other hand, a smartphone provided by the health management system has recently been attracting attention. Smartphones or mobile phones are convenient and accessible and suitable to present age when the demand for PHRs and smart medical technology are important [15], enabling various opportunities for health care. In addition, the use of the latest mobile technologies called mobile health or mHealth is increasing in popularity in the medical field, which has become an important advancement [16]. Therefore, high-risk mothers are the most suitable target group for applying mHealth because of its optimal functions.

We believe that health management information service protocols in the entire pregnancy of high-risk mothers are important; thus, we propose the development of a health application protocol through a smartphone to promote better health behavior.

## Methods/design

### Objectives

The overall goal of this trial is to target the effectiveness of a smartphone app for health care interventions in high-risk mothers. Providing healthcare interventions via a smartphone app demonstrates the effectiveness of smart medical technology. Therefore, in this study, more specifically, the study group using the app and the group using paper health management information will compare over a period of 6 weeks.

### Study design

**Fig. 1:**
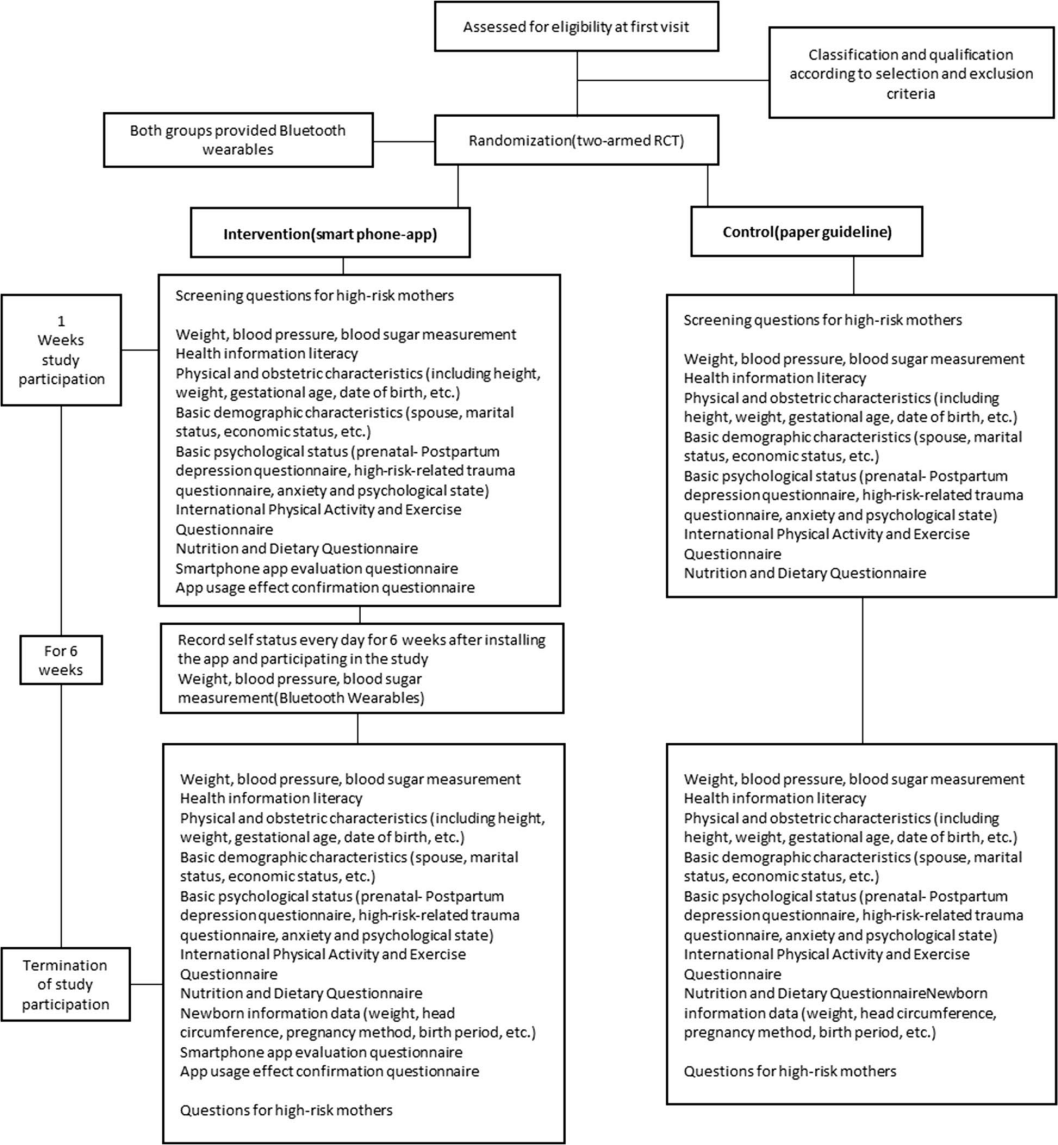
Trial flow diagram

This study will be conducted in accordance with SPIRIT’s clinical trial protocol guidelines. This study will be conducted as a clinical trial study according to the following framework and will compare the two groups. This randomized controlled trial (two-armed RCT) will be conducted for 6 weeks to provide healthcare service information based on a smartphone app for high-risk mothers during their entire pregnancy. A diagram of the study design is shown in Fig. 1. In addition, based on the research design, the procedure for developing a mobile app using the IDEAS framework [17] was followed, which is an appropriate procedure to lead to healthy and positive outcomes through the development of mobile digital medical interventions. The procedure is as follows [18] (Table 1):

**Table 1:** List of 10 phases of the IDEAS framework

1	Empathize with target users
2	Specify target behavior
3	Ground in behavioral theory
4	Ideate implementation strategies
5	Prototype potential products
6	Gather user feedback
7	Build a minimum viable product
8	Pilot test to assess potential efficacy and usability
9	Evaluate efficacy in randomized controlled trial
10	Share intervention and findings

### Ethics

This study is in accordance with the WMA Declaration of Helsinki—Ethical Principles for Medical Research Involving Human Subjects. Also, this study received institutional ethics approval from the Gachon University Gil Hospital in South Korea in January 2022.

### Enrollment and eligibility

This study will be conducted at the Department of Obstetrics and Gynecology and Public Medical Center of Gachon University Gil Hospital in South Korea. This hospital is affiliated with the university and is in charge of medical care for the entire pregnancy cycle. Women aged ≥ 18 years who were diagnosed with gestational diabetes, gestational hypertension, and depression during or after pregnancy will recruit if they own a smartphone.

Participants selected by the medical staff will be admitted to the hospital. After that, you will be informed of consent and explanation for participation in the study by the research staff. Written or verbal informed consent will be obtained from all participants. Personal and demographic information, including contact information, will be collected. Also, On the consent form, participants will be asked if they agree to use of their data should they choose to withdraw from the trial. Participants will also be asked for permission for the research team to share relevant data with people from the Universities taking part in the research or from regulatory authorities, where relevant. This trial does not involve collecting biological specimens for storage. Upon randomization, participants will be categorized into two groups (intervention and control groups). Next, all participants will receive a Bluetooth weighing machine, blood pressure machine, and wearable blood glucose machine.

### Allocation of participants

The participants will be randomly assigned in the intervention group applying the app and the general control group providing the paper guidelines performed using computer-generated assignments in a 1:1 ratio. Considering the characteristics of the intervention, both the participants and researchers will aware of the group to which they will be assigned. In addition, this study will be conducted as a stratified block. Accordingly, the registration of participants and the assignment of interventions will be performed by the medical staff, and a random number table will be generated through a computer program to assign and enroll participants in the study.

### Sample size

Since this study is not testing a statistical hypothesis, but rather exploratory, it is decided to proceed with the minimum number of subjects empirically required within the limit that satisfies the purpose of the study. In general, it should be considered that such exploratory research is usually conducted with around 50 people. Most high-risk pregnant women do not want to visit the hospital after childbirth, so when obtaining consent in advance, patients who have actively consented to long-term observation should be registered. Therefore, it was determined that 12-month observation data could be collected from 150 subjects when about 300 subjects were recruited by calculating the dropout rate as about 50%. Separately, this study checked the health of mothers and newborns before and after pregnancy and considered that most complications such as gestational diabetes occurred within 2 years after childbirth. Accordingly, this study is continuously necessary for a total of 3 years, about 1 year including the entire period of pregnancy, and 2 years to confirm the occurrence of complications and childbirth results as a follow-up. Therefore, about 150 women will receive relevant clinical data every year, and 500 mothers in total will be selected as target subjects.

### Control arm

Participants assigned to the control group will receive paper guidelines about diseases, appropriate exercise during pregnancy, proper diet and nutrition, and pregnancy and newborn care information.

### Intervention

We will randomize participants to the intervention installed on the app via their smartphone. Mothers using this app measured their weight, blood sugar, and blood pressure every day, and the Bluetooth wearable device automatically entered the values into the app. Through this measured and recorded information, the patient will send an intervention message by push notification according to all pregnancy cycles. The intervention message will be delivered to check the nutritional information and exercise method according to the numerical value, and the mother acquires health information while checking the content. In addition to providing medical information throughout the pregnancy cycle, mothers will also receive a schedule of tests to be performed during pregnancy. By the time the mothers will give birth, they will have healthcare information about newborn care and postpartum disease prevention. With this information, mothers can take healthy precautions that benefit them throughout pregnancy. Furthermore, these healthy behaviors can help prevent diseases in the postpartum period.

### Intervention content

The intervention content will be used in the app consists of evidence-based guidelines [19–24] for mothers and literature search materials. For evidence-based data on gestational hypertension, gestational diabetes, and prenatal-postpartum depression, two obstetricians and gynecologists conducted an Agree-II evaluation to confirm the quality and level of evidence. This item will be decided. In particular, exercise during pregnancy is recommended to maintain stability, according to the RCOG guideline [25]. The items agree on the content of the arbitration, as shown in Table 2 below. In addition, the message will be provided in line with the timing of the pregnancy cycle is a bomb push notification so that the detailed items belonging to the major items will be rotated twice a week to reduce the fatigue of mothers.

**Table 2:** Intervention content and push message to offer pregnancy cycle interventions

**Main items**	**Main detail items**	**Intervention message**	**The timing of provision according to the pregnancy cycle**
**Delivery**	Prevention education related to childbirth	Hi, $NAME$! Here are some tips to help you prepare for a healthy birth to meet your upcoming baby!	A week before the scheduled date, after delivery, or can be read when clicked
	Postpartum blood pressure management	Hi, $NAME$! If you are worried about your blood pressure readings after your blood pressure test, read these resources! (Normal value: 120/80 mmHg)	Once a week after delivery, or can be read when clicked
	Postpartum proteinuria care	Hi, $NAME$! If proteinuria is detected as a result of a urine test, read the following materials!	Once a week after delivery, or can be read when clicked
**Medical information**	Management of postpartum hypertension	Hi, $NAME$! Let me tell you how to manage gestational hypertension, how to manage postpartum hypertension, how to manage it, risk factors for recurrence, and information that helps prevent recurrence!	record daily figures and Send Information notification, Can be read when clicked
	Recurrence prevention guide	Hi, $NAME$! Let me give you information that helps prevent recurrence of gestational hypertension and recurrence risk factors!	Can be viewed at click
	Chronic Hypertension Risk and Prevention Factors	Hi, $NAME$! Let me show you the risk factors related to hypertension and the prevention factors of hypertension!	Can be viewed at click
	Relevance to cardiovascular disease	Hi, $NAME$! Here’s a guide to the link between high blood pressure and cardiovascular disease and the long-term risk factors!	Can be viewed at click
	Guide to hospitals with gynecologists stay in	Hi, $NAME$! We will tell you about the basic measures needed in an emergency, as well as nearby emergency centers and obstetrics and gynecology hospitals!	Can be viewed at click
	Prenatal and postpartum depression	Hi, $NAME$! If you are experiencing ups and downs in your emotions and moods due to sudden hormonal changes, read these resources!	record daily figures and Send Information notification, Can be read when clicked
**Newborn care**	Newborn development information	Hi, $NAME$! How well is my baby growing? Find out how your baby’s average body changes by week with following data!	A week before the scheduled date, after delivery, or can be read when clicked
	Newborn health care and caring	Hi, $NAME$! What does our baby eat, how much it grows, how does it need to be washed? Find out how to manage newborn health through the following data!	A week before the scheduled date, after delivery, or can be read when clicked
	Newborn crisis management	Hi, $NAME$! If our baby suddenly gets sick or has strange symptoms, how do we deal with it? Find out how to deal with the crisis through the following data!	A week before the scheduled date, after delivery, or can be read when clicked
**Diet and nutrition**	Basic information on diet and eating habits	Hi, $NAME$! What kind of eating habits should have to take care of your health? Learn about your eating habits that can improve your health through the following data!	After participating in the study, detailed items below diet and nutrition are sent twice a week. And can be read when clicked
	Information on salt intake and its dangers	Hi, $NAME$! How much salt should we consume per day? How much sodium is in your diet? Find out with these resources!	After participating in the study, detailed items below diet and nutrition are sent twice a week. And can be read when clicked
	Healthy eating habits	Hi, $NAME$! It is very important to maintain normal blood sugar, blood pressure, and weight through good eating habits. Find out what eating habits are helpful through the following data!	After participating in the study, detailed items below diet and nutrition are sent twice a week. And can be read when clicked
	Nutrition and recommended foods	Hi, $NAME$! You should take enough nutrients during pregnancy. Find out about essential nutrients and food through the following data!	After participating in the study, detailed items below diet and nutrition are sent twice a week. And can be read when clicked
	Nutrient ratio	Hi, $NAME$! Did you know that there are five major nutrients to be consumed during pregnancy? Find out the five nutrients you need from you through the following data!	After participating in the study, detailed items below diet and nutrition are sent twice a week. And can be read when clicked
	Attentional food	Hi, $NAME$! Find out what foods you should be careful about because they are not good for pregnancy!	After participating in the study, detailed items below diet and nutrition are sent twice a week. And can be read when clicked
	Nutritional supplements by gestational period	Hi, $NAME$! What are additional nutritional supplements other than nutritional intake through food, and when should I take them? Find out more about the nutritional supplements you need to take by pregnancy period through the following data!	After participating in the study, detailed items below diet and nutrition are sent twice a week. And can be read when clicked
	Morning sickness management	Hi, $NAME$! What is the occasional morning sickness during pregnancy, and how can it be relieved? Find out how to take care of morning sickness through the following resources	After participating in the study, detailed items below diet and nutrition are sent to the message twice a week. And can be read when clicked
**Exercise and physical activity**	The physical activity guide	Hi, $NAME$! Excessive exercise during pregnancy has a risk of premature birth. Find out about the appropriate amount of physical activity and exercise for mothers through the following resources!	After participating in the study, detailed items below exercise and physical activity are sent to the message twice a week, And can be read when clicked
	Guide to the intensity and type of exercise	Hi, $NAME$! Exercise is necessary for effective weight, blood pressure, and blood sugar management. Find out more about the types and intensity of exercise to help you get pregnant with these resources!	After participating in the study, detailed items below exercise and physical activity are sent to the message twice a week, And can be read when clicked
	Restrictions and precautions for exercise and physical activity	Hi, $NAME$! Exercise is necessary for effective weight, blood pressure, and blood sugar management. Learn more about exercise and physical activity precautions during pregnancy with these resources!	After participating in the study, detailed items below exercise and physical activity are sent to the message twice a week, And can be read when clicked
	The importance of weight management	“Hi, $NAME$! Read our resources on how to change and manage your weight properly for your desired weight management!	After participating in the study, detailed items below exercise and physical activity are sent to the message twice a week, And can be read when clicked
	Information on weight change during pregnancy	“Hi, $NAME$! Find out what is proper weight change during pregnancy and how weight changes for pregnant women in general!	After participating in the study, detailed items below exercise and physical activity are sent to the message twice a week, And can be read when clicked
	Weight management after childbirth	Hi, $NAME$! Read our article on proper weight change and how to manage it for good postpartum weight management!	After participating in the study, detailed items below exercise and physical activity are sent to the message twice a week, And can be read when clicked
	Prenatal and postpartum exercise guide	Hi, $NAME$! Prenatal-postpartum exercise is a great help to your physical and mental health, and is also essential for rapid recovery. Find out the importance, type and recommended amount of prenatal-postpartum exercise!	After participating in the study, detailed items below exercise and physical activity are sent to the message twice a week, And can be read when clicked
**Self-record**	A daily log of your condition during pregnancy	Hi, $NAME$! It’s a self-record for me! Write every day on the subject this week! You don’t have to write long. Just write at least three sentences!	Send a message once a week, or can be read when clicked
**Emergency/urgency management**	Crisis of gestational hypertension	Hi, $NAME$! In the case of gestational hypertension, a hypertensive crisis can strike unexpectedly. Find out what a hypertensive crisis is and how to deal with it with these resources!	Send a message once a week, or can be read when clicked
	Guide on risk blood sugar level	Hi, $NAME$! What is gestational diabetes and what is the blood sugar level that you diagnose as diabetes? Find out the blood sugar levels of gestational diabetes through the following data!	Send a message once a week, or can be read when clicked
	Guide on depression symptoms and examination	Hi, $NAME$! The hormone changes that occur during pregnancy also affect your mood and feelings. With deep depression or negative emotions, check your psychological state with the following data!	Send a message once a week, or can be read when clicked
	Hospitals where you can receive information in case of an emergency	Hi, $NAME$! How can you deal with an unexpected crisis during pregnancy? Find out the following data about the possible crisis during pregnancy and how to do it!	Send a message once a week, or can be read when clicked

### Intervention channel: smartphone app

Mothers randomly assigned to the intervention group will download the app. It will be available free of charge on Android-based and Google Play. This app fills in one’s gestational age, expected delivery date, basic information about the disease, exercise amount, and dietary pattern during registration. By pushing a message through this app, one can acquire health information and promote one’s own health behavior. The study participants will be received a message programmed to push notifications every day. The items configured in Table 2 were pushed as messages according to the pregnancy cycle. Mothers will receive the health information that is needed to check via push notifications or will be viewed by clicking on it.

Additionally, mothers will be required to access and engage the app on a daily basis. In particular, blood pressure, blood sugar, and weight were recorded. These daily records can provide a cautionary warning when risk levels are reached during pregnancy, which can prevent emergencies.

This app has five core functions.

1. Mothers can record their blood sugar, blood pressure, and weight every day and check the changes through calendar images and self-manage whether the blood sugar, blood pressure, and weight can be managed at appropriate and maintain them at normal levels.
2. The section on physical activity and exercise is made with evidence-based data as practical tips during pregnancy and is composed of image and video files for easier use. It consists of physical activities and exercises that can be performed in daily. Messages about participation and promotion will send twice a week to prevent fatigue by pushing the same content.
3. Comprehensive guidelines for weight, nutrition, and diet during pregnancy have been formulated based on evidence-based data. A more suitable dietary pattern and nutritional diet will be provided based on the first dietary pattern recorded by the person.
4. A validated Edinburgh Depression Test Sheet was used for prenatal-postpartum depression. Mothers will be guided to re-test once every two weeks, and a notification will appear so that you can check the mother’s mood and emotions once a week. Additionally, questionnaires about anxiety, maternal–fetal attachment, self-esteem, social support, and traumatic experience in high-risk pregnancies were provided so that more specific psychological states will be identified.
5. One can make a self-record. Mothers can self-record their condition during pregnancy. Previous research has showed that self-recording can promote changes in one’s own behavior and self-esteem and that it helps reduce physical symptoms, such as blood pressure.

The smartphone app key features are to enable mothers in acquiring the pregnancy’s health care information, showing that high-risk mothers can change into healthy mothers with better behaviors towards health. The main functions of this app were prepared based on evidence from various overseas guidelines and literature data and were confirmed by obstetricians, gynecologists, and expert councils to suit the characteristics of high-risk mothers. In addition, clinical psychologists and preventive medicine scientists, including app design companies, presented opinions on establishing detailed data. In addition, the usage, main functions, and each item of the participant’s app users will be recorded and quantified when the study is completed.

### Outcome measure

Table 3 shows the intervention and control groups measured item results, and the same items will be measured after the 6-week study participation period. Primary measurements will include weight, blood pressure, blood sugar and health literacy, and physical and obstetric characteristics. In addition, character, exercise and nutritional information on basic demographic characteristics and basic psychological state will be collected. For the primary measurement, the same information will be collected for both the primary and final results for comparison with the final result.

**Table 3:** Outcome measure items

1	Weight, blood pressure, blood sugar measurement
2	Health information literacy
3	Physical and obstetric characteristics (including height, weight, gestational age, date of birth, etc.)
4	Basic demographic characteristics (spouse, marital status, economic status, etc.)
5	Basic psychological status (prenatal-postpartum depression questionnaire, high-risk-related trauma questionnaire, anxiety, and psychological state)
6	International Physical Activity and Exercise Questionnaire (IPAQ)
7	Nutrition and Dietary Questionnaire
8	Newborn information data (weight, head circumference, pregnancy method, birth period, etc.) (after 6 weeks/in case of delivery)
9	Questions for high-risk mothers
10	Smartphone app evaluation questionnaire (only intervention group)
11	App usage effect confirmation questionnaire (only intervention group)
12	Self-report (only intervention group)

Items 1 to 9 in Table 3 are for the intervention and control groups measurement. Item 1 measures body weight, blood pressure, and blood sugar daily during the study period. The amount of change from the start to the end of the study will be evaluated. Item 9 is about high-risk mothers and asks them to check for the diseases that apply to them, such as gestational hypertension, gestational diabetes, and prenatal-postpartum depression. This item will be completed at the beginning and end of the study. When the study is over, it will also be checked whether the disease is maintained. This can help with the prevalence and maintenance of healthcare-related diseases.

On the other hand, to check changes in health behaviors, the International Physical Activity Questionnaire (IPAQ) [26] is used to evaluate changes in health behaviors with a questionnaire verified in the pregnancy cohort. In addition, the results of maternal nutrition and diet measure the change in health behavior toward diet and nutrition by comparing before and after the mother’s recorded eating habits and nutrition and dietary behavior questionnaires.

### Statistical analysis

This study will compare the outcomes between the two groups using IBM SPSS software for statistical analysis. For this purpose, descriptive statistics and correlation analysis of each group are performed, and a paired sample T-TEST of two groups is performed, so that comparative analysis can be performed. In addition, pre-post-group comparison analysis will be conducted, especially for both groups, from the first outcome to the final outcome. Hierarchical and stepwise regression analyzes will be conducted to determine the impact of each item to look at changes in health behavior as the end result.

## Discussion

This randomized controlled trial aimed to change the health behavior of high-risk mothers by providing health care information services through the development of a smartphone app. This research can be said to be the safest and most suitable research in the present, where smart medical technology is considered more important than mHealth technology. In addition, a smartphone app can be used by anyone and anywhere, promoting a healthier and more adaptive life and playing a big role in preventing diseases.

Therefore, this study will significantly help mothers and newborns in maintaining a healthy life during and after pregnancy. In addition, if this app is used properly in actual clinical sites and the general community, this smartphone app is expected to play a very useful role for medical staff. The accumulated information acquired through this app can be of help to medical staff in determining more efficient diagnosis and treatment management, which can contribute to the formation of medical artificial intelligence algorithms.

However, considering that the actual clinical setting or the characteristics of a high-risk mother are a more complicated process through various situations and changing physical conditions, this smartphone app will not replace the medical staff in the actual field. Nevertheless, there is no doubt that this app will be a great tool for medical staff, guardians, and patients to collaborate by playing an auxiliary role in a healthy life and prevention of disease via innovative medical technology. In the future, it will be necessary to establish a continuous algorithm through collaboration between obstetricians and gynecologists, preventive medicine, psychologists, and IT experts, and to discuss the direction of use and connection of smart medical technology. In addition, smartphone-based apps using the currently configured items will require clinical verification through various studies, including actual clinical studies, to secure external validity in the future [27]. Therefore, empirical research or analysis of this smartphone application should be conducted in follow-up studies.

## Trial status

Current protocol version: March 2022. This trial has not yet started.

It is anticipated to start on July 01, 2022.

Anticipated end date for recruitment: According to the approval of the ethical institution, this study will end in December 23rd, and recruitment will end if the number of study subjects is recruited.

## Dissemination policy

The authors will disseminate their findings by publishing one or more articles in peer-reviewed journals in the relevant field, based on the data and materials from this study.

## Data Availability

The authors will disseminate their findings by publishing one or more articles in peer-reviewed journals in the relevant field based on the data and materials from this study. In addition, final study protocol data can be accessed by all authors, and final study results can be created. If there is a request for a dataset and protocol set, it can be provided with the consent of the research funding organization and the researcher. All research-related materials can be provided with the consent of the research funding institution and the researcher.
